# Madelung’s Disease Evolving to Liposarcoma: An Uncommon Encounter

**DOI:** 10.3390/life14040521

**Published:** 2024-04-17

**Authors:** Mihaiela Lungu, Violeta Diana Oprea, Gabriela Stoleriu, Ana-Maria Ionescu, Andrei Lucian Zaharia, Ana Croitoru, Bianca Stan, Elena Niculet

**Affiliations:** 1 Faculty of Medicine and Pharmacy, “Dunarea de Jos” University of Galati, 800008 Galati, Romaniazaharia.andreilucian@gmail.com (A.L.Z.); croitoruana28@yahoo.com (A.C.);; 2 “St. Ap. Andrei” Clinical County Emergency Hospital, 800579 Galati, Romania; 3 “St. Spiridon” Clinical Emergency County Hospital Iasi, 700111 Iasi, Romania; 4 Faculty of Medicine and Pharmacy, Ovidius University of Constanța, 900470 Constanța, Romania; iuliusana@gmail.com

**Keywords:** multiple symmetric lipomatosis, benign symmetric adenolipomatosis, alcoholism, cervical compression, liposarcoma, Launois–Bensaude syndrome, Madelung’s disease, malignant transformation

## Abstract

(1) Background: Madelung’s disease—known also as Benign Symmetric Adenolipomatosis (BSA) or Multiple Symmetric Lipomatosis (MSL), is a rare subcutaneous tissue disease characterized by the proliferation of non-encapsulated fat tissue with mature adipocytes. Patients develop symmetrical fatty deposits of varying sizes, (located particularly around the neck, shoulders, upper and middle back, arms, abdomen, and thighs), having clinical, esthetic, and psychiatric repercussions. (2) Methods: We report a case diagnosed with BSA upon admission to the Neurological and Internal Medicine Departments of the Emergency Clinical Hospital of Galati. (3) Results: This patient developed compressive phenomena and liposarcoma with liver metastasis, followed by death shortly after hospital presentation. The histopathology examination confirmed right latero-cervical liposarcoma and round cell hepatic metastasis. The specific metabolic ethiopathogenic mechanism has not been elucidated, but the adipocytes of BSA are different from normal cells in proliferation, hormonal regulation, and mitochondrial activity; a rare mitochondrial gene mutation, together with other interacting genetic or non-genetic factors, have been considered in recent studies. A thorough literature search identified only three cases reporting malignant tumors in BSA patients. (4) Conclusions: The goal of our paper is to present this rare case in the oncogenic synergism of two tumors. In the management of this BSA disorder, possible malignant transformation should be considered, although only scarce evidence was found supporting this.

## 1. Introduction

Madelung’s disease (MD), also named Benign Symmetric Adenolipomatosis (BSA)/Multiple Symmetric Lipomatosis (MSL) or Launois–Bensaude syndrome, is a rare metabolic syndrome characterized by symmetrical deposition of subcutaneous adipose tissue in the head, neck, shoulders, back, trunk, and nerve roots of the upper and lower limbs [1,2,3,4]. The first cases were described by Brodie in 1846 [5], and systematically summarized and discussed by Madelung in 1888 [6] followed by Launois and Bensaude in 1898 [7]. To date, not many cases of BSA have been described in the medical literature [8,9,10,11,12], of which we were only able to identify three other cases that were associated with a related malignant tumor [13,14,15].

This rare lipid metabolic disorder has a prevalence of 1:25,000; it usually affects men much more frequently than women (a male/female ratio estimated at around 15/1 up to 30/1), and is more common around the Mediterranean basin [1,2,3,4]. Nevertheless, there are reports of large study groups where the male/female ratio is reversed—for example, from a German cohort, Schiltz et al. reported a M/F ratio of 1:2.5 [16], while Plummer at el. found a 1:6 ratio in their seven-case series [17].

Most BSA cases appear to be isolated, but there are also reports of familial aggregation, with autosomal dominant inheritance [18].

The onset often occurs between 30 and 60 years of age, although the literature shows cases of much earlier onset, from childhood or teenage years [10,11].

A large proportion of patients are alcoholics and research linked chronic alcohol abuse to an accelerated progression of the disease [13,18]. It is hypothesized that one of the pathogenic mechanisms is alcohol damaging adrenergic lipolysis by affecting enzyme processes in mitochondria, thus acting as a cofactor inducing a change in the number and function of b-adrenergic receptors [18,19,20,21].

Adipogenesis is considered to be caused by an exaggerated hyperplastic proliferation of the subcutaneous brown adipose tissue. The pathogenesis of BSA involves generating new adipocytes rather than the expansion of existing cells.

Genetic pathogenesis has also been discussed, starting from the evidence of mutations presented by a minority of patients having large-scale deletions and specific point mutations within mitochondrial DNA (mtDNA), as well as in some nuclear genes encoding mitochondrial proteins, such as MFN2 encoding mitofusin-2 [22,23,24].

The disease manifests through the initial fast development of non-encapsulated fatty masses—containing mature adipocytes—that generates symmetrical deposits of varying sizes (2 to 20 cm). They usually form around the neck, face, occipital region, in the clavicular fossae, around the shoulders but they can also appear at the level of the thorax, abdomen, and thighs [25]. Therefore, the old classification of the disease has changed over the last few years [17].

**Figure 1:**
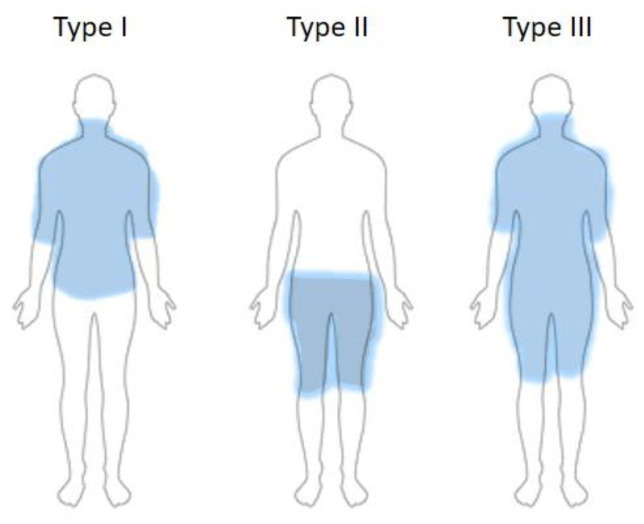
Different phenotypes of the new Schiltz classification (2018), showing distribution of the symmetrical fatty masses [17].

Classification of BSA is mainly based on clinical criteria describing the different phenotypes. It evolved over time from two clinical types to three main types (Schiltz et al. [17]); see Figure 1.

Before Schiltz et al. proposed their new classification system based on a German patient cohort in 2018 [17], the were two main classifications used in the literature. Enzi et al. (E) [26,27] described two types of anatomic distribution of fat deposits. Type 1 (E) is predominantly distributed in the neck (“Madelung’s collar”), shoulders, supraclavicular triangle, and proximal upper limbs. In type 2 (E), the neck area and upper trunk are not affected; deposits occur in the abdomen and thighs.

A widely used classification was described in 1991 by Donhauser (D) dividing patients into four types of BSA [28]:

- Type I (Madelung horse collar)—localization of fatty masses in the cervical region, upper arms.
- Type II (Launois–Bensaude “pseudo-athletic type”)—affected body areas are upper arms, thorax, deltoid region; it does not appear to be connected to alcohol abuse.
- Type III (“gynecoid type”)—fat tissue masses located in the lower body, especially thighs and internal knee side.
- Type IV (“abdominal type”)—abdominal lipomatosis.

Because some patients with multiple symmetric lipomatosis could not be included in Donhauser’s three classes, Schiltz et al. carried out a study between 2007 and 2017 on 45 BSA patients [17], resulting in a new classification of different phenotypes:

- Type Ia—fatty masses around the neck, corresponding to type I (D)—found in 3% in their study group.
- Type Ib—fatty masses on the neck, shoulders, and arms, corresponding to pseudoathletic type II (D)—in 4% of their cohort.
- Type Ic—as per Type Ib, but also including the trunk (new to Donhauser classification).
- Type II—lipomatosis in the lower part of the body, thighs, and pelvis, corresponding to type III (D).
- Type III—generalized disposition of the fatty masses, excluding the head, forearms, and lower legs.

Over time, fat tissue masses can lead to more than just esthetic changes, such as compression of the carotid arteries in the latero-cervical region, the trachea, and the esophagus, some resulting in respiratory obstruction and sleep apnea [14,25]. In addition, in time many patients can develop depression due to body shape alterations. However, it remains unclear whether the depression or mood changes can be directly related to BSA, as there are no studies on the association between mental health and the disorder [29,30,31].

BSA can be associated with neuropathies; myopathies; reduced deep tendon reflexes; paresthesia; hypothalamic and pituitary lesions; neuroendocrine syndromes; sudden death; autonomous nervous system dysfunctions; myoclonic epilepsy with ragged red fibers (MERRF); and carotid compression [11,17,32,33,34,35]. Other associated manifestations include liver failure; metabolic syndrome; altered glucose tolerance; dyslipidemia; lipodystrophy; and obesity—most patients are overweight, while around 10% of the cases have normal weight [25,26,27,32,33]. Hypercholesterolemia, hypothyroidism, and hyperinsulinemia and insulin resistance, (comorbidities often found in BSA patients), suggest a possible link to metabolic diseases [27,36,37].

We found no clear evidence that BSA directly impacts life expectancy, but long-term follow-up studies showed high rates of incidences of somatic neuropathy and sudden death due to fat occupation in the mediastinal space [30,31]. In a 12-year follow-up study of one patient, Suito et al. reported death from hemorrhagic shock due to hepatocellular carcinoma and hepatorenal syndrome, although they found no recurrence of fat masses [32].

The aim of this paper is to report a rare case of an oncogenic synergism of two concurrent tumors. Only a few cases of the fatty tissue masses becoming malignant have been presented in the literature; we were able to identify articles reporting the onset of liposarcoma or intramyxoid sarcoma in just three patients suffering from BSA [14,15,16,38,39,40,41]. Our case-based literature review aims to draw attention to malignant transformation of lipomatous lesions in BSA and compare data from the available literature, adding new information on histologic alterations in the course of the disease.

## 2. Materials and Methods

We report a patient case diagnosed with BSA upon admission to the Neurology and Internal Medicine Departments of the Emergency Clinical Hospital of Galati, Romania. The diagnosis was clinically and histologically confirmed, using Schiltz classification and light microscopy of paraffin-embedded tissue, stained with hematoxylin and eosin performed on necrotic exam.

The literature search was conducted in the PubMed and Google Scholar databases, using combinations of the relevant selected keywords: multiple symmetric lipomatosis; benign symmetric adenolipomatosis; Launois–Bensaude Syndrome; Madelung’s disease; liposarcoma; malignant transformation.

The study was approved by the Ethics Committee of the St. Ap. Andrei Clinical County Emergency Hospital, Decision no. 19787/17.09.2020. Written informed consent for the patient’s data use for the study, histopathologic preparation of the tissues, and publication of the relevant data was obtained from the family of our deceased patient.

## 3. Results

**Figure 2:**
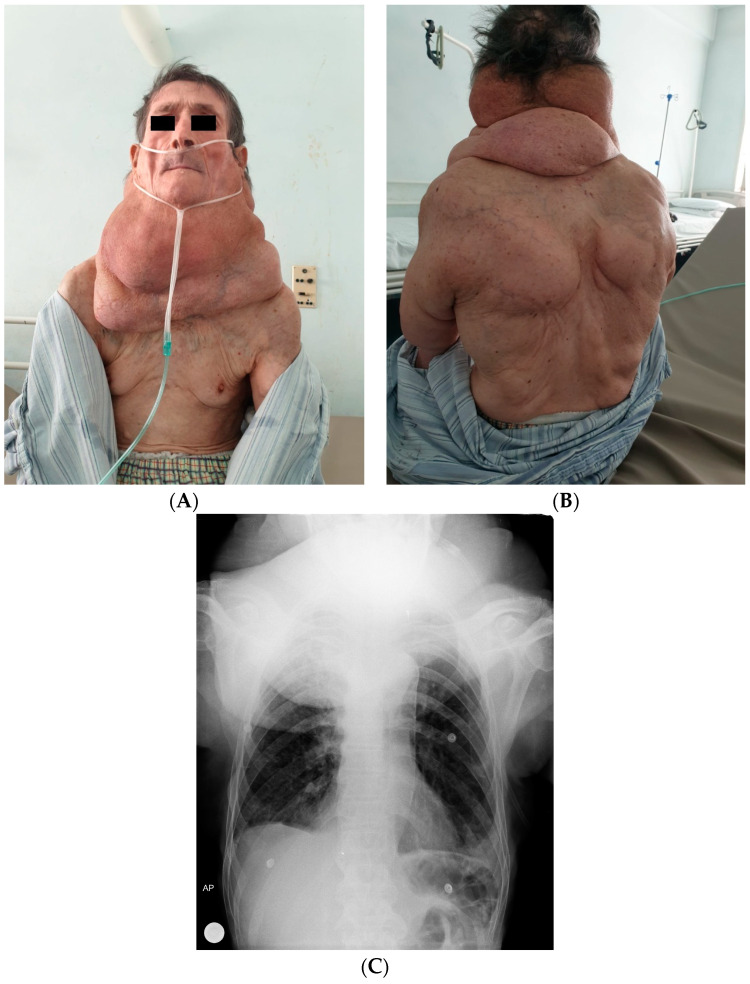
Large adenolipomatosis masses, in the cervical, thoracic, and arms regions: (**A**) front picture; (**B**) back picture; (**C**) X-ray of the thorax.

Our 74-year-old patient was admitted to the Internal Medicine Department for severe progressive dyspnea and significant weight loss occurring over the previous 4–5 months. Personal history revealed chronic alcohol abuse (up to 8 units of alcohol per day, for over 15 years) and the existence of fatty masses localized in the cervical and thoracic regions and on the arms (Figure 2A,B). The case was classified as a type Ic Multiple Symmetric Lipomatosis, according to Schiltz et al. or type II Donhauser.

The onset of growing fatty masses was recalled by the patient’s family as being at the age of ~20 years old and having progressively grown in size.

Clinical examination showed several large lipoma-looking lesions compressing the structures around the cervical region. The patient presented anterior thorax collateral vessels suggestive of the compression of the vena cava. On the right lateral cervical region within the fat tissue, a hard painless nodular 10 cm lesion, mobile on the deep layers, was identified. The liver reached 6 cm below the right rib, and had a hard rounded edge, the patient being cachectic. A malignant transformation in liposarcoma was suspected in the right laterocervical tumoral mass.

A thoracic X-ray showed a left tracheal deviation (Figure 2C). Lab testing revealed hepatic cytolysis, with an increase in serum alkaline phosphatase and an elevated erythrocyte sedimentation rate.

Sadly, the patient passed away 24 h after admission to the hospital, so no further imagistic investigations could be performed.

**Figure 3:**
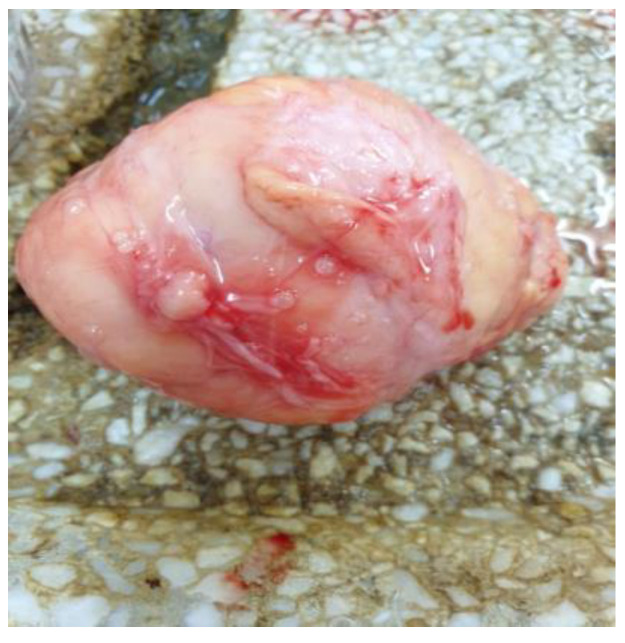
Autopsy examination: a big nodular tumor (liposarcoma) from the right latero-cervical region, resembling normal fat except for the uncharacteristic fibrous bands.

The autopsy confirmed the large lipomas, revealed the cervical malignant nodular lesion (Figure 3) and showed a necrotic liver metastasis with no other secondary lesions.

A histopathology exam of the tumors confirmed the multiple adenolipomatosis, a right latero-cervical well-differentiated liposarcoma (WDL), and a big necrotic liver metastasis.

**Figure 4:**
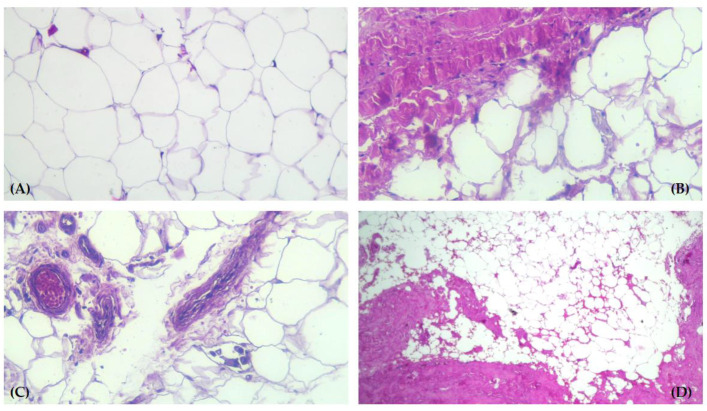
Well-differentiated liposarcoma—histopathology examination. (**A**) Numerous lipoblasts with triangular-shaped hyperchromatic nuclei. Light microscopy of paraffin-embedded tissue, stained with HE ×100. (**B**) Prominent fibrous areas and lipoblasts in the tumor structure. Light microscopy of paraffin-embedded tissue, stained with HE ×100. (**C**) Thickened fibrous septa containing collagen fibers of varying thickness and large hyperchromatic nuclei. Light microscopy of paraffin-embedded tissue, stained with HE ×100. (**D**) Fibrous septa and hyperchromatic nuclei, showing the malignant tissue in the vicinity of blood vessels. Light microscopy of paraffin-embedded tissue, stained with HE ×40.

A microscopic examination of the WDL described the tumor mass as predominantly composed of mature adipocytes, a variable number of atypical fusiform cells with hyperchromatic nuclei, and lipoblasts. These lipoblasts were multivacuolated, containing lipid droplets in the cytoplasm. The fibrous areas within the tumor structure consisted of trabeculae traversing the adipose tissue; they contained collagen fibers of different thicknesses, in which spindle cells and multipolar stromal cells with hyperchromatic nuclei were found. These cells were also present among the mature adipocytes in the tumor structure (Figure 4A–D).

**Figure 5:**
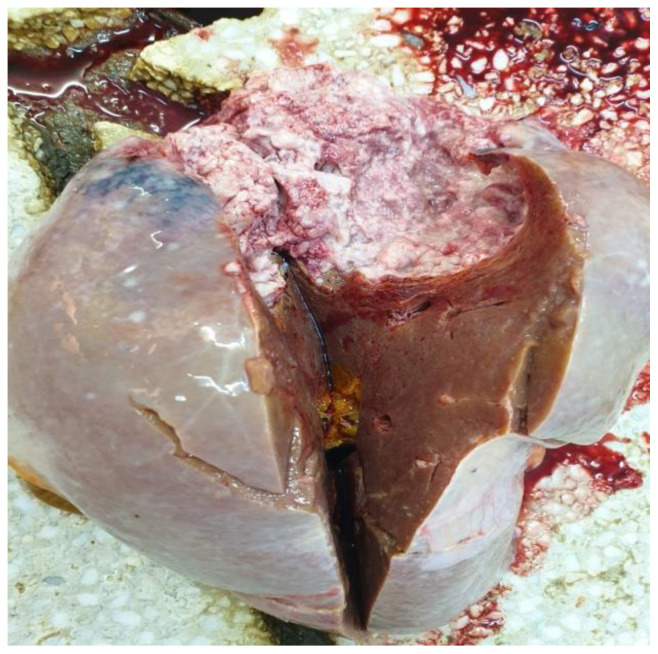
Liver necrotic nodular metastasis—necrotic exam, macroscopic aspect: pale areas of tissue damage.

**Figure 6:**
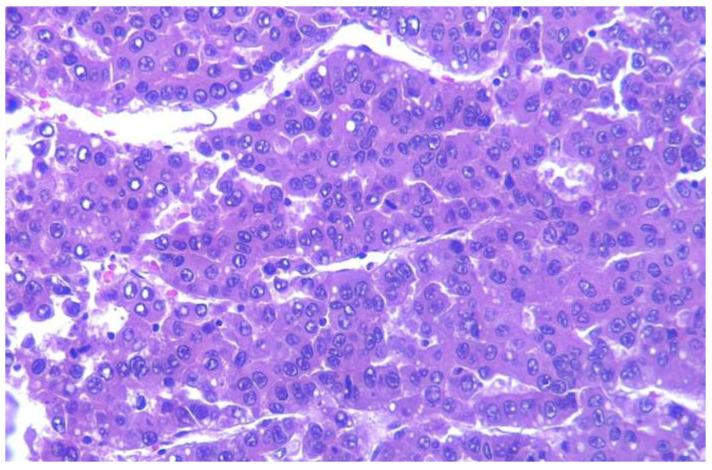
Light microscopy of paraffin-embedded liver metastasis, stained with HE ×40.

The liver metastasis was a nodular 12/8 cm tumor, with pale areas of tissue on the surface of the section (Figure 5). The microscopic examination of the liver tumor samples confirmed the diagnosis of liposarcoma metastasis (Figure 6).

## 4. Discussion

### 4.1. Pathogenesis of BSA

BSA’s pathogenesis is not fully understood yet; defects in the mitochondrial noradrenergic regulation involving brown fat are under consideration.

A mitochondrial cytopathy with point mutations at the ragged red fiber syndrome locus MERRF (myoclonus epilepsy and ragged red fibers), or human mitochondrial MNF2 genes have been implicated in pathogenesis. Genetic forms have also been described, including mutations in the MFN2 gene (1p36.22) and LIPE gene (19q13.2), which code mitofusin 2 (MFN2) and hormone-sensitive lipase (LIPE), respectively [34,35,36,37]. Additionally, mitochondrial and cAMP dysfunctions are involved. Family history research is also needed [19,20,21,22,23,24,25].

Klopstock et al. identified mtDNA mutation in BSA patients without a history of alcoholism, but in none of the patients with high alcohol intake [24]; in a different study, Pasmatzi did not find mitochondrial DNA m.8344A > G mutation in BSA [37].

Human immunodeficiency virus 1 (HIV1)-positive patients treated with protease inhibitors can manifest a Madelung’s disease-like condition with diffuse proliferation of the subcutaneous fat [42,43,44].

As in our case we could not identify any family history of similar conditions, and a genetic assessment postmortem was not available, we cannot confirm any involvement of a gene mutation in this patient.

### 4.2. Diagnosis of BSA

Clinical appearance is usually suggestive enough for BSA with no other patient complaints except for esthetic issues. Ujpal and Nemeth evaluated the medical literature between 1998 and 2000, discovering 190 cases, with a wide spread of the disease around the Mediterranean Sea [25]. The large adipose masses (2–20 cm) are weakly delimited within the adjoining tissues, have soft consistency, and are non-encapsulated (so over time they tend to infiltrate the adjoining regions). Histologically, they contain mature adipocytes forming normal fat tissue [45].

Ultrasonography is the first choice for imagistic confirmation of BSA, being accessible and widely available. CT scans or MRI analysis confirm, alongside the biopsy, the definite diagnosis [41,45,46]. It was demonstrated in long-term surveillance studies that for soft tissues tumors MRI provides superior diagnostic accuracy with high sensitivity and specificity, also offering valuable details that support therapy and monitoring for recurrencies [47,48,49].

Unfortunately, in our patient’s situation further imagistic investigations were not possible due to fast aggravation evolving to death by cardiopulmonary arrest, soon after hospital admittance.

### 4.3. Differential Diagnosis, Comorbidities, and Malignant Transformation in BSA

A differential diagnosis should be made with: Cushing syndrome; partial familial Dunnigan lipodystrophies (determined by mutations in the LMNA gene); familial angiolipomatosis; simple obesity; neck cysts; lymphoma; leukemia and soft tissue sarcoma; spiradenocarcinoma; and salivary and thyroid gland disease, is necessary [37,38,39,40,41,45,46,50,51]. A biopsy can exclude malignancy or prove the concomitant presence of liposarcoma or an intramyxoid sarcoma [10,14,15,52,53,54,55,56,57,58,59,60]. Liver disease is a common comorbidity of BSA, and acute renal failure may also occur [10,20,24,25,26,27,28,50,51]. In non-alcoholic type 2 BSA, a pseudoathletic appearance may be misdiagnosed as obesity. The differential diagnosis may also include other conditions such as familial multiple lipomatosis; Cushing’s syndrome; iatrogenic cutaneous lipomatosis; encapsulated lipomas; angiolipomatosis; myxoid liposarcoma; and lymphoma [2,10,11,12,41,45,46]. An essential diagnostic tool for BSA disease, where available, is computed tomography with multiplanar reconstruction (MPR) or volumetric rendering technique (VRT), allowing evaluation of the severity of the lesions and planning of a therapeutic schedule.

A biology lab profile may indicate high-atherogenic mixed dyslipidemia, high basal insulinemia (30 microU/mL), and multiple markers of insulin resistance (Reaven index; lipid accumulation product; homeostatic model; insulin sensitivity index; and modified glycemic curve following oral glucose load) [61,62,63,64,65].

Other associated conditions are noted, such as obesity; diabetes; central and peripheral neuropathy; myopathy; macrocytic anaemia; dyslipidemia; hyperuricemia (gout) and hypothyroidism; arterial hypertension; chronic obstructive pulmonary disease; alcoholic fatty liver or cirrhosis; and hepatopathy [61,62,63,64,65,66]. Malignant tumors developed in BSA are uncommon and have been reported in only three cases until now, concerning liposarcoma or intramyxoid sarcoma [13,14,15,37,38,39,40,41].

In general, the medical literature states that lipomas cannot transform into liposarcomas, although some papers attempted to prove this based on histopathology and clinical evidence [13,14,15,37,38,39,40,41]. Another perspective of our case would be the one of liposarcoma presenting concomitant BSA as an association of two tumors, within an oncogenic synergism [67,68,69,70]. The two tumors, BSA and liposarcoma, could develop differently from a histopathological point of view, but the localization of the malignant tissue within the benign mass does not provide clear evidence in support of their distinction. Well differentiated liposarcomas may be underdiagnosed as lipomas, as they may have histopathologic variation; in addition, large lipomas are not always separated and analyzed into many different areas. The microscopic diagnosis for this type of liposarcoma is related to the identification of atypical stromal cells rather than the identification of lipoblasts [41,54,55,56,69,70,71,72,73]. It is possible that further extended cytogenetic analysis of differentiated lipomatous lesions will allow a more precise evaluation of this problem.

### 4.4. Therapy of BSA

Treatment is necessary due to esthetic or physiological reasons (compression of respiratory and digestive structures). Manual lymphatic drainage before the fibrosis of the fat tissue, physical therapy, low calorie diet, and skincare are useful during the onset of the disease. Surgical treatment consists of ultrasound assisted resection, liposuction, or lipectomy, but relapses are reported [47,74].

Some studies show that use of salbutamol (β_2_-adrenergic agonist) 12 mg per day can slow down the progression of the disease, by stimulating lipolysis [75].

Fibric acids (PPARα agonists), as fenofibrate 200 mg daily, can improve the BSA condition. Growth hormone (GH) replacement must be considered only for those who are GH-deficient. Local injections with corticosteroid, thyroxine, enoxaparin, deoxycholate, and phosphatidylcholine have been suggested as a treatment for lipomatosis such as BSA [53,54,55,56,57,58].

PBSerum^®^ containing bioactive enzymes—Hyaluronidase PB3000 (an enzyme that breaks down polysaccharides from excessive concentrations responsible for fluid accumulation); Collagenase CoL GH PB2200 (the enzyme that dissolves adipose nodules in advanced cellulite); and Lipase PB500 (the enzyme that induces the adipocyte lyse)—proved to provide fast results, but its use is limited to localized adipocyte deposits at their onset [44,52,53,54,55,56,76].

Recent advances in mitochondrial gene therapy as well as mitochondrial replacement therapy with in vitro fertilization may offer potential future management options. In addition, coenzyme Q10 supplementation was suggested to have a benefit, given its role as a mitochondrial electron transport chain stabilizer [61,62].

Correct treatment of comorbidities is essential.

Genetic advice is necessary, because there is evidence of genetic pathogenesis—autosomal (recessive or dominant) defects or those associated with mitochondrial genetic mutations.

Alcohol abstinence is recommended, but studies proved it does not lead to a reduction in the fatty masses.

The prognosis of BSA is influenced by comorbidities. Although it is a benign condition, the illness consists of white adipogenesis upregulating AKT, CK2 and ERK1/2 [55,56] and can have important esthetic and psychological repercussions [47,70,74] or result in death by sleep apnea [75]. Because central nervous system involvement can occur, (many authors have reported the presence of neuropathy in about 85% of patients with BSA and an association with sudden cardiac death), it is important to closely monitor for the presence of neurological symptoms.

## 5. Conclusions

BSA is a rare disorder of adipocyte differentiation, characterized by benign, diffuse, symmetrical lipomatosis; our literature review identified less than 400 cases (including available meta-analyses) but only three presenting malignant transformation.

In our case the clinical appearance was classified as a type Ic multiple symmetric lipomatosis, according to Schiltz et al. or type II Donhauser. This patient developed compressive phenomena, malignancy (liposarcoma) with liver metastasis, followed by death soon after late hospital presentation. Alcohol consumption was probably a contributing factor in the aggravation of the disease. The patient did not report any neurological symptoms, even though BSA presented in the medical literature usually associates them.

The aim of our work is to present the rarity of this particular case in the oncogenic synergism of two tumors with similar histopathological origins. Considering the extreme rarity of BSA transforming into malignant tumors, we consider reporting such a case both intriguing and clinically meaningful. In the management of BSA, the possible association with a malignant tumor should ultimately be considered, and pathologists should carefully look for WDL in excisional biopsy specimens.

## Data Availability

The data presented in this study are available on reasonable request from the corresponding authors.
